# Early-onset hereditary isolated non-neurogenic orthostatic hypotension in a Swedish family

**DOI:** 10.1007/s10286-023-00963-9

**Published:** 2023-07-17

**Authors:** Jan Fagius, Joakim Klar, Niklas Dahl

**Affiliations:** 1 Department of Medical Sciences/Neurology and Clinical Neurophysiology, Faculty of Medicine, Uppsala University, Uppsala, Sweden; 2 Department of Immunology, Genetics and Pathology/Genetics, Faculty of Medicine, Uppsala University, Uppsala, Sweden

**Keywords:** Orthostatic hypotension, Autonomic nervous system, Microneurography, Autosomal dominant hereditary disorder

## Abstract

**Purpose:**

Orthostatic hypotension is a common condition with heterogeneous and, in many cases, unclear underlying pathophysiology. Frequent symptoms are syncope and falls with a strong impact on daily life. A two-generation family with eight individuals segregating early-onset severe orthostatic hypotension with persistent tachycardia in upright position and repeated faints was identified. Our aim was to elucidate the underlying pathophysiology.

**Methods:**

One severely affected individual underwent thorough investigation with neurophysiological and blood pressure (BP) measurements, including direct recording of baroreflex-governed sympathetic nerve signalling and induction of BP rise with phenylephrine. Family members underwent parts of the examination. Genetic analysis using exome sequencing was performed.

**Results:**

Marked postural hypotension with greatly reduced cardiac preload was observed, but without signs of autonomic nervous system dysfunction: sympathetic nerve signalling was normal, as were catecholamine levels, and phenylephrine stimulation revealed a normal increase in BP. The results of the genetic analysis using exome sequencing comprising all known genes associated with the regulation of BP and catecholamine metabolism were normal.

**Conclusion:**

The combined findings suggest an autosomal dominant form of early-onset orthostatic hypotension with variable clinical expression and without any additional autonomic dysfunction. It is possible that further investigation will reveal an as yet undescribed entity of orthostatic hypotension transmitted as an autosomal dominant trait.

**Supplementary Information:**

The online version contains supplementary material available at 10.1007/s10286-023-00963-9.

## Introduction

The upright body position in humans is a challenge, not only in terms of being able to balance and maintain this position with respect to gravity, but also in terms of blood pressure (BP), with the risk of a fall in BP with the change from a supine to upright position [1]. The autonomic nervous system (ANS) is a potent regulator of the BP acting to meet this challenge. Disorders involving the ANS may cause a postural fall in BP with faint and, in severe cases, marked orthostatic intolerance and neurogenic orthostatic hypotension [2]. Orthostatic hypotension despite an intact ANS may occur rapidly with blood loss or dehydration, whereas chronic non-neurogenic hypotension may be caused by adrenal insufficiency, heart disease with reduced output, antihypertensive treatment and excessive vasodilation [2]. A related phenomenon is postural orthostatic tachycardia syndrome (POTS), which was initially described as “sympathotonic orthostatic hypotension” [3] but subsequently established that the marked tachycardic reaction in the upright position characteristically occurs without hypotension [1, 4, 5].

Orthostatic hypotension (OH) has been formally defined [1, 6] as a reduction in systolic BP of at least 20 mmHg or a reduction in diastolic BP of at least 10 mmHg within 3 min of standing. Emphasis has been placed on the fact that OH is a physical sign and not a disease. A rapid brief fall in BP an active change to the upright position has been labelled “initial orthostatic hypotension” [1, 7, 8] and defined as a systolic BP reduction of > 40 mmHg.

A number of neurological disorders of central (pure autonomic failure, multiple system atrophy) and peripheral (polyneuropathy, predominantly diabetic and amyloid; to a lesser degree hereditary) origin may cause autonomic dysfunction with OH [9, 10]. Among the genetic diseases resulting in OH, familial dysautonomia (HSAN III, Riley-Day syndrome) holds an exceptional position, with severe autonomic dysfunction and a multitude of other neurological symptoms [11]. Hereditary disorders associated with a disturbed biosynthesis or release of noradrenaline, the main transmitter in the regulation of the sympathetic vascular tone and circulation, give rise to autonomic dysfunction by inhibiting the intact nerve signals from reaching their effector organs. [12–14].

While an understanding of such diseases has brought important information on mechanisms underlying OH, the specific causes of severe and early-onset OH forms remain elusive in many cases. Identification of familial cases with the disturbance may therefore open up for new insights into the regulation of orthostasis. In this framework, we report on a hereditary disorder, which to our knowledge has not previously been described, with extreme and rapidly evolving orthostatic intolerance without any signs of ANS pathophysiology and with preserved catecholamine levels. The disease showed an autosomal dominant inheritance pattern with variable expressivity.

All subjects described herein provided informed consent to the publication of detailed data. The study was approved by the Swedish Ethical Review Board (Dnr 2021–03384) and conformed to the standards of the 1964 Declaration of Helsinki and its later amendments.

## Family members

**Fig. 1:**
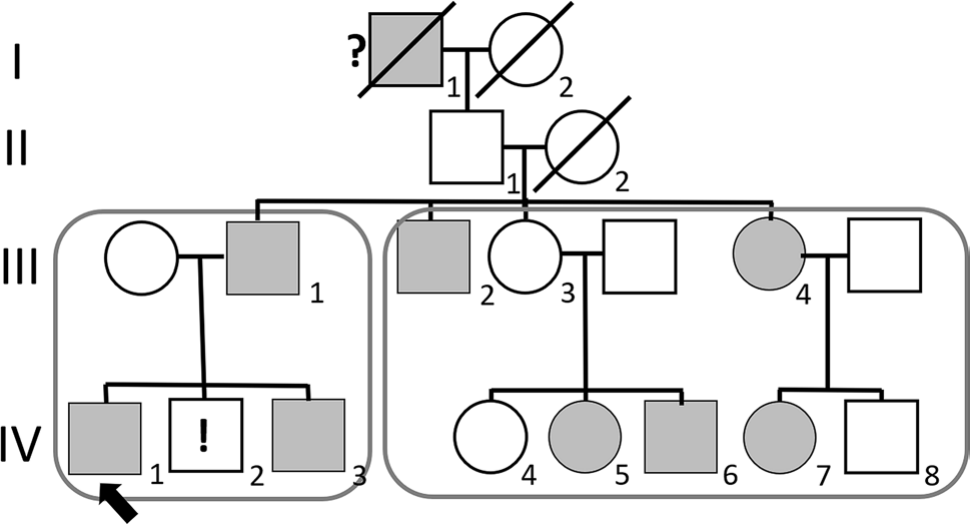
Pedigree of the extended family demonstrating autosomal dominant orthostatic hypotension (OH). The index case (*IV:1*, arrow) belongs to the key family (box on left) with the most severely affected members; current close relatives in generations III and IV are depicted in box on the right. Grey filled boxes and circles denote affected individuals. Subjects II:1 and III:3 showed no symptoms of OH, suggesting reduced penetrance. Question mark denotes that OH was not ascertained from the clinical investigations but strongly suggested based on anamnestic information. Exclamation mark indicates asymptomatic subject with clinically recorded OH. See text for more details. Diagonal line is standard in pedrigrees to indicate deceased subjects

The family under study originates from northern Sweden and spans four generations (Fig. 1). During the years 1999–2000 the index patient (IV:1) and key family members (subjects III:1, IV:2 and IV:3) underwent thorough investigations after referral to the Department of Neurology, University Hospital, Uppsala (Sweden). Other close relatives in generation III and IV (Fig. 1, right box) who were reported to have similar problems in the upright position were examined less extensively by one of the authors (JF) at local primary care clinics in northern Sweden.

Specific details on the key family under study and ancestors are as follows:

- *Subject I:1* was reported by the descendants to have been suffering from a young age from episodes of dizziness when rising up and sometimes syncope; details not known.
- *Subject II:1* is alive, aged 93 years, but symptoms are not reported in this intermediate generation.
- *Subject IV:1* (index patient) was born after normal gestation and delivery. From the age of 10 years onwards he noticed a tendency for repeated fainting when rising and standing that increased in severity with age. At age 15 years he suffered from marked orthostatic intolerance, and OH was documented. Innumerable faints occurred, associated with paleness, palpitations and sometimes cold sweat, indicating at least partially intact autonomic functions. The patient was 20 years old at referral. He was tall (193 cm), with a body mass index (BMI) of 18.8 kg/m^2^. A physical examination revealed no abnormality except for OH (see below) and excavated feet. He constantly experienced mild lightheadedness when standing still, and he could not rise abruptly from lying to standing without a risk of fainting. There were periods of exaggerated orthostatic intolerance lasting days through weeks. He reported normal urinary bladder and sexual functions, normal sweating and no heat intolerance.
  Polyneuropathy had initially been suspected due to pes excavatus but was not confirmed at repeated clinical and neurophysiological examinations. The patient had tried dihydroergotamine, salt supplementation and fludrocortisone as treatment for OH, without any effect of practical benefit. Compressive garments (knee stockings) brought about some subjective improvement but no obvious increase in tolerance to rapid postural challenge.
- *Subject IV:3*, the youngest brother of the proband, was referred for clinical evaluation at the age of 17 years. Similar to his above-mentioned brother, he is slim and tall for age (194 cm; BMI 16.1 kg/m^2^) with excavated feet from birth. He was otherwise healthy until the age of 14 years, when he successively developed a strong tendency to faint in the upright position. Bouts of exaggerated orthostatic intolerance occurred, which on a few occasions were reversed by rapid infusion of Ringer’s solution. The orthostatic intolerance, together with other seemingly intact autonomic functions, was phenomenologically identical to that of the index patient.
- *Subject IV:2* is the middle and healthy brother without postural symptoms. He is less slim than his brothers but slightly taller (196 cm, BMI 25.0 kg/m^2^), and foot configuration was normal. He was referred at the age of 21 years as part of the family investigation.
- *Subject III:1* is the father of the proband and was referred at the age of 53 years as part of the family investigation. At the examination, he exhibited a similar slender and tall body constitution (192 cm, BMI 23.1 kg/m^2^) as his sons, and he had experienced lightheadedness and occasional fainting from late adolescence onwards. The orthostatic intolerance varied, as for the sons, sometimes from one day to another. No detailed examination related to OH had been conducted prior to the present referral. The orthostatic intolerance was never as severe as that of his sons. Bladder and sexual functions were intact.

The close relatives in generation III and IV (Fig. 1, right box) were examined in the autumn of 2021 and are described in brief in the following section.

## Methods

A number of routine circulatory examinations on the key family members were performed at Uppsala University Hospital, as described in the “Results” section. The most extensive investigations were performed on the index patient. The following additional special physiological investigations were performed by author JF.

### Direct recording of sympathetic nerve signals

**Fig. 2:**
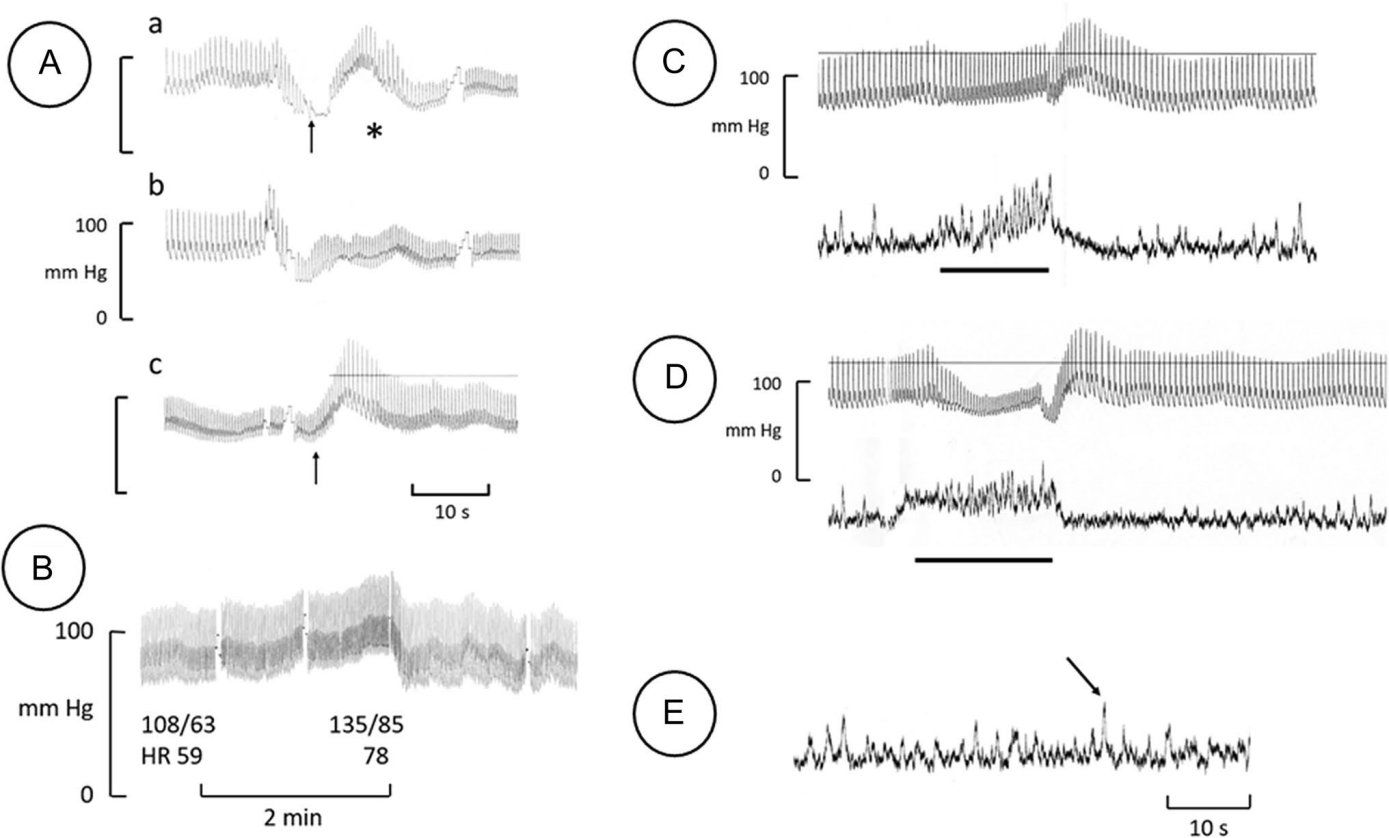
Blood pressure (BP) reactions (**A**–**D**), muscle sympathetic nerve activity (MSNA;** c**,** D**: lower traces) and skin sympathetic nerve activity (SSNA) (**E**) recorded in index patient IV:1.** B**–**E** Index patient in supine position. **A** BP reactions in patient when lying and standing: *a* Rapid rise from lying position, showing presyncope (arrow), sitting down for a few seconds, rising anew (asterisk);* b* slower rise (4–5 s) from lying, showing lightheadedness, paleness, eye deviation (see text);* c* standing with low BP; crossing legs (arrow). Same time scale is shown in* a*–*c*. **B** Continuous BP record during 2 min of sustained handgrip at 33% of maximal strength. Numerical values represent BP (top values) and heart rate (*HR*) during preceding rest and at end of manoeuvre. Note qualitatively normal rise in BP and tachycardia during the manoeuvre. **C** MSNA (lower trace) and BP during 17 s of apnoea (horizontal bar). Each peak in the neurogram corresponds to a burst of baroreceptor-governed sympathetic nerve signals. Note normal marked increase in MSNA followed by an increase in BP during apnoea, and normal inhibition of MSNA during the post-apnoea BP rise. **D** MSNA and BP during a 20-s Valsalva manoeuvre (horizontal bar). Normal reaction with strong increase in MSNA during the manoeuvre, turning the lowering of BP to an increase, and normal profound inhibition of MSNA during the post-manoeuvre BP rise with a return to habitual resting level first after approximately 40 s. **E** SSNA of normal appearance during silent rest. Each peak in the neurogram corresponds to a burst of sympathetic (sudomotor/vasoconstrictor) nerve signals. The strongest burst (arrow) was provoked by a slight slap on the patient’s nose, illustrating normal arousal sensitivity of SSNA. Breaks in BP recordings were due to automatic calibration of equipment. Faint upper horizontal line in** A**(c),** C** and** D** is due to printer irregularities

Microneurography was performed in muscle and skin fascicles of the right peroneal nerve [15, 16]. Muscle sympathetic nerve activity (MSNA) displays cardiac rhythmicity and contributes to BP regulation by inhibitory baroreflexes. Skin sympathetic nerve activity (SSNA) lacking an obvious relation to the heart rhythm is involved in short-term body temperature regulation. SSNA is also very sensitive to arousal [15, 16]. In the current recordings a moderate signal-to-noise ratio was obtained (compare Fig. 2c, D).

### Blood-pressure distorting manoeuvres with noninvasive continuous BP monitoring

These manoeuvres were performed using a finger cuff (Finapres technology; Ohmeda, Englewood, CO, USA [17, 18]) during active rising to an upright position following at least 3 min of supine rest, the Valsalva manoeuvre, sustained hand grip (1/3 of maximal strength) for 2 min and end-expiratory apnoea (with the two latter causing a transient rise in BP through an increase in MSNA [19, 20]). During the rise to a standing position, the hand holding the Finapres device was fixed at heart level; during the other manoeuvres, the hand lay at the bed level. The device also delivers instantaneous heart rate (HR) and numerical BP values every third second.

### Intravenous infusion of phenylephrine during continuous BP monitoring

The infusion was administrated by stepwise dose increase every 6 min (0.2, 0.4, 0.8, 1.6, and 2.4 μg/kg/min, with the last dose given for only 3 min in order to avoid induction of a hypertensive reaction). The final dose was followed for 6 min post-infusion. The BP was monitored as described above (Finapres technology).

### Exome sequencing

Exome sequencing was performed on genomic DNA extracted from peripheral blood of index patient IV:1. A QIAsymphony extraction robot (Qiagen, Hilden, Germany) was used to obtain DNA, and whole-exome sequencing (WES) was performed using TWIST comprehensive exome according to the manufacturer’s protocols (Twist Target Enrichment Protocol; Twist Bioscience, South San Francisco, CA, USA). The library was sequenced on a NovaSeq SP flowcell using paired-end 100-bp read length and v1 sequencing chemistry (Illumina, San Diego, CA, USA). Sequencing reads were aligned to the hg19 reference sequence followed by variant calling and annotation. We used the bioinformatic bcbio-nextgen pipeline version 1.1.5 (https://pypi.org/project/bcbio-nextgen/), including the bwa 0.7.17, picard-tools-1.96, samtools 1.9 and gatk 4.1.3.0 software packages. Copy number variation (CNV) analysis was performed using ExomeDepth v1.1.15 using unrelated samples as a reference set [21]. The generated VCF-files were analyzed with the Franklin software from genoox (https://franklin.genoox.com/clinical-db/home) using the Human Phenotype Ontology (HPO) terms ‘Abnormality of habitus’, ‘Polyneuropathy’ and ‘Postural hypotension’ and the phenotype ‘orthostatic intolerance’, ‘pes cavus’ and ‘venous insufficiency’ to search for pathogenic or known disease associated gene variants. Variants presenting > 1% in the Genome Aggregation Database (gnomAD) v2.1.1 were excluded from analysis. Special attention was given to the analysis of genes encoding proteins of the catecholamine metabolism (e.g. tyrosine hydroxylase (*TH*), aromatic l-amino acid decarboxylase (*DDC*), dopamine β-hydroxylase (*DHB*), cytochrome B561 (*CYB561*) and phenylethanolamine* N*-methyltransferase (*PNMT*) pathways.

### Examination of the additional family members

Three siblings of subject III:1 (i.e. III:2–4; Fig. 1) and their five offspring in generation IV (i.e. cousins to index patient IV:1) underwent a brief clinical examination (by JF) testing neurological functions and BP in supine and upright positions in the autumn of 2021.

## Results

### Subject IV:1 (index patient)

Clinical examination of subject IV:1 (Fig. 1) was normal with the exception of the presence of marked OH and the foot configuration without signs of peripheral neuropathy. Chest and palate were normal (i.e. no signs of Marfan syndrome). His sweat moistness in hands and feet were normal (i.e. mildly moist but not wet). There was no distension of the venous system or blueish discolouration of the feet, as sometimes reported in persons with POTS [5, 22].

**Fig. 3:**
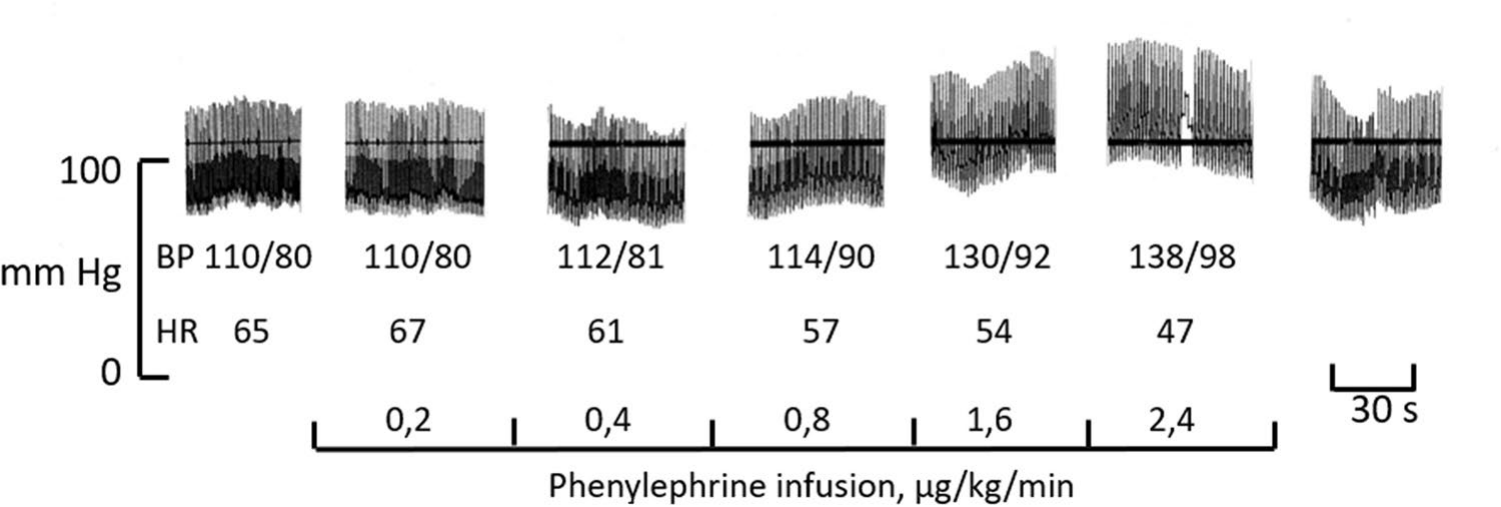
Blood pressure and HR of index patient in response to a stepwise increase in doses of intravenous phenylephrine. The bottom row of numbers indicates the doses; the duration of each step was 6 min, except for the final dose, which lasted for 3 min. The upper horizontal line due to printer malfunction Top: Segments of continuous intra-arterial BP recordings at end of each dose (break in 1 BP segment is due to automatic calibration of equipment); the same time scale was used in all segments. The BP measurements are the mean of 5 blood numerical pressure values at the end of the dosing period. The HR measurement is the mean of 5 instantaneous HR measurements taken at the end of the dosing period. Note the dose-dependent rise in BP, indicating normal response to catecholamine stimulation, as well as dose-dependent bradycardia, indicating normal baroreceptor inhibition of HR with increased BP

The results of the further testing of the index patient are as follows:

- Neurography, electromyography and sensory thresholds were normal.
- Autonomic test results according to clinical signs, including sweating detected by galvanic skin response (GSR; electrodermal response), palmar and plantar skin vasoconstriction (photoelectric plethysmography; van Gogh, Amsterdam, Netherlands) and RR-interval variation during deep breathing and Valsalva, were normal.
- Routine BP measurements revealed a supine resting BP of 127/73 mmHg, which was 96/78 mmHg after 2 min of standing upright. The corresponding HR were 61 and 118 beats per minute (bpm), respectively. BP measurements were obtained from the following tests:

- Routine tilt test according to the “Italian protocol” [23] revealed a pronounced fall in BP with immediate more than doubling of HR (54 to 128 bpm); administration of nitroglycerine caused faint with unmeasurable BP.
- Active rise to standing position with continuous BP recording showed showed a similar drop in BP and increased HR. Typical BP reactions with continuous BP measurement are shown in Fig. 2A. Leg crossing with the thighs pressed together at standing [24] strongly reduced the fall in BP (Fig. 2A part c). The BP and HR changes, measured using the Finapres device, varied with rising speed from the lying to standing position (Table 1). A normalization of BP, HR and subjective feeling was always achieved within 5–10 s after resuming a recumbent position. Table 1 also shows BP reactions after he administration of oral midodrine, 60–90 min after stepwise dose increases of 2.5 to 5–10 mg, as well as the marked stabilizing effect of BP by standing with crossed legs; no effect on BP was recorded in standing position after midodrine administration.
- Test cycling in the sitting position showed a marked increase in HR (56–184 bpm), a weak and slow systolic BP reaction (105–130 mmHg), but for age a good working capacity; the patient reported slight light-headedness. Test cycling in lying position brought about normal increases in both HR (56–159 bpm) and systolic BP (100–159 mmHg); no light-headedness.
- BP reaction to sustained handgrip during 2 min was qualitatively normal in both the lying and sitting position, with an increase in BP and HR (Fig. 2B).
- BP reactions to apnoea and Valsalva manoeuvres were both qualitatively normal (Fig. 2c, D; a detailed description is provided below).

- Ultrasound cardiography was normal in the recumbent position. In a sitting position, slightly bent forward, a markedly reduced preload was noted, without significantly reduced BP, thus suggestive of a low venous return.
- Colour Doppler ultrasonography of leg veins showed normal anatomy, ordinary valves and no sign of leakage. A similar Doppler ultrasonography of carotid and vertebral arteries revealed no abnormality.
- Abdominal computed tomography (lying position) did not reveal any abnormal dilatation of pelvic veins (abdominal organs normal as well).
- Direct recording of sympathetic signals was performed with microneurography in the peroneal nerve. MNSA and, after electrode adjustment, SSNA, were recorded. Both types of activity occurred in the respective normal burst pattern: MSNA bursts displaying cardiac rhythmicity and an inverse relationship to BP fluctuations (Fig. 2c, D); SSNA bursts in an irregular pattern, with clear arousal response (Fig. 2E). MSNA outflow (burst frequency) at supine rest was 30 bursts/min (somewhat higher than mean outflow in subjects of the patient’s age [25, 26]. Apnoea induced an increase in MSNA with subsequent rise in BP (Fig. 2c), and a Valsalva manoeuvre brought about a normal pattern (Fig. 2D), i.e. a reduction in BP inducing an initial increase in MSNA, followed by an increase of BP during the ongoing manoeuvre, and a final inhibition of sympathetic outflow with normal post-manoeuvre rebound rise in BP. Strength of individual bursts, measured as mean amplitude (mm) in the neurogram, increased 59% during apnoea and 39% during Valsalva.
- Serum noradrenaline level at rest was slightly above the upper normal limit (mean of 2 analyses: 2.25 nmol/L; reference interval: 0.7–2.1), with a normal increase after standing up (mean of two analyses: 3.2 nmol/L). A likewise normal noradrenaline response was observed at repeated testing performed 2 years later.
- Other catecholamine analyses, dopamine, diurnal cortisol curve, serum aldosterone, urine osmolality, plasma renin, plasma troponin-I, myocardial perfusion scintigraphy, isotope angiography, continuous electrocardiogram (ECG) for 48 h, carotid massage and blood volume measurement were all within normal limits (see Electronic Supplementary Material [ESM] file 1 for details).
- Phenylephrine infusion induced a stepwise increase in BP that was clearly related to the stepwise rise in dose (Fig. 3), indicating normal noradrenergic receptor reaction to catecholamine stimulation. The phenylephrine-induced increased BP was accompanied by a likewise dose-dependent reduction in HR (Fig. 3). The patient reported no discomfort during the infusion.
- Bioinformatic analysis of exome sequencing data revealed no pathogenic gene variants. All detected gene variants are listed in ESM Table S1, file 2. Variants shortlisted by the Franklin software using the relevant HPO terms were excluded based on either the inheritance pattern (recessive genes), absence of reported pathogenicity (benign or likely benign in ClinVar) or high population frequency (ESM Table S2, file 3).

**Table 1:** Blood pressure and heart rate reactions within 15–30 s after active rise from supine to upright position in index patient IV:1

Treatment	Active rise	Lying (BP; HR)	Standing	Standing (symptoms/signs^a^)
No treatment	Rapid rise	104/64 mm Hg; 65 bpm	86/67 mm Hg; 125 bpm	Syncope New attempt: presyncope
	Semirapid rise	106/60 mm Hg; 63 bpm	91/68 mm Hg; 125 bpm	Presyncope
	Slow rise	109/63 mm Hg; 60 bpm	96/74 mm Hg; 125 bpm	Dizziness
	Rise to standing with crossed legs	101/76 mm Hg; 63 bpm	120/107 mm Hg	0
1.5 h after administration of midodrine 10 mg	Rapid rise	112/67 mm Hg; 45 bpm^c^	78/61 mm Hg; 110 bpm	Syncope New attempt: presyncope
	Slow rise	116/70 mm Hg; 45 bpm	77/54 mm Hg; 105 bpm	Dizziness
	Rise to standing with crossed legs^b^	116/70 mm Hg; 45 bpm	108/72 mm Hg; 105 bpm	0

Each numerical value is the mean of 8–10 consecutive values displayed every third second by the device used

BP Blood pressure, bpm beats per minute, HR heart rate

^a^Presyncope: paleness, gaze deviation upwards, protruded lips. New attempt: sitting a few seconds, then a new rise

^b^BP with crossed legs is the stable level following a brief initial higher value; c.f. Figure 2A, part c

^c^Supine bradycardia after the administration of midodrine is assumed to be secondary to the vasopressor effect of the drug

### Subjects III:1 and IV:3

Both subjects had a body constitution similar to that of the index patient, including pes cavus. The results of the clinical examination were otherwise normal, with the exception of markedly low BP when standing (Table 2). There were no clinical signs of polyneuropathy, and sweating was normal in hands and feet*.*

**Table 2:** Blood pressure and heart rate reactions within 15–30 s after active rapid rise from supine to upright position in symptomatic patients III:1 and IV:3

Subject	Lying (BP; HR)	Standing (BP; HR)	Standing (symptoms/signs)	Sustained standing (BP; HR)^a^
III:1	110/61 mm Hg; 76 bpm	61/34 mm Hg; 129 bpm	Presyncope, pale	90/69 mm Hg; 108 bpm
IV:3	94/76 mm Hg; 74 bpm	52/44 mm Hg; 135 bpm	Presyncope, swaying, pale	85/74 mm Hg; 125 bpm

^a^Sustained standing means standing for at least 2 min

The results of further testing of subjects III:1 and IV:3 are as follows:

- Neurography, electromyography and sensory thresholds (performed due to foot configuration) were normal.
- Routine autonomic testing, performed as for subject IV:1, was normal in both subjects.
- BP reactions:

- Rapid active rise to standing position with continuous BP recording (Table 2) revealed an abnormal pattern similar to that of the index patient and with a twofold increase in HR (see Fig. 2A). The effort was associated with presyncope and paleness. Standing with crossed the legs gave a marked rise in BP and freedom from orthostatic symptoms in subject IV:3 (compare with Fig. 2A, part c); this manoeuvre was not tested for subject III:1. Recovery after return to lying was rapid in both cases.
- Routine tilt test revealed a pronounced fall in BP to unmeasurable values with a 57% increase in HR in the younger brother (subject IV:3) whereas the test was within normal limits for the father (this test not done the same day as active rise to standing).
- BP reaction to apnoea and Valsalva manoeuvre was qualitatively normal, as for the index patient (compare with Fig. 2c, D).
- Test cycling in sitting position (case IV:3) showed marked increase in HR (68–182 bpm), with a normal systolic BP increase (105–182 mmHg) and good working capacity; no light-headedness. (Not performed for case III:1.)
- Carotid massage did not evoke fall in BP.

- Occlusion plethysmography in lower arm with infusion of nitroprusside and metacholine in subject IV:3 was normal, i.e. with no sign of nitric oxide-dependent abnormal vasodilatation. (This test not done in the index patient and subject III:1).
- Serum noradrenaline level at rest and after standing up, urine excretion of catecholamines, serum adrenaline and cortisol levels (morning and afternoon) were normal in both subjects.
- An attempt to direct recording of sympathetic nerve activity in case IV:3 failed for technical reasons.

### Subject IV:2

This brother was tall but not as slender as his father and two brothers. He exhibited somewhat high foot arches, but not as marked as the others. Results from both the physical and clinical neurological examinations were normal, as were those of neurography and electromyography. A limited number of tests was performed:

Active rise to standing position with continuous BP recording evoked a marked fall in BP and sustained tachycardia, but no symptoms (Table 3). With return to lying there was a rapid BP recovery as in the above cases. Routine autonomic testing, tilt test, BP reaction to apnoea and Valsalva manoeuvre and reaction to carotid massage were all normal, as were catecholamine and cortisol levels.

**Table 3:** Blood pressure and heart rate reactions within 15–30 s after active rise from supine to upright position in asymptomatic subject IV:2

Active rise	Lying (BP; HR)	Standing (BP; HR)	Sustained standing (BP; HR)^a^
Rapid rise	116/68 mm Hg; 72 bpm	71/35 mm Hg; 110 bpm	95/65 mm Hg; 104 bpm

^a^Sustained standing means at least 2 min

### Further course of subject IV:1 and IV:3

The index patient was initially helped in daily life by crossing his legs when standing. A propranolol attempt (which may improve POTS due to β-receptor hypersensitivity [27]) was of no value, whereas midodrine and fludrocortisone in combination gave some subjective improvement. Over time there were bouts of exaggerated orthostatic intolerance–sometimes with, but often without, any infection or other overt deconditioning event–requiring hospitalization; treatment consisted of rapid infusion of Ringer’s solution [28] and stepwise training of orthostatic tolerance under a physiotherapist’s supervision. During a telephone follow-up at age 40 years he described occasional bouts of severe orthostatic symptoms over the last decade. He was wheelchair bound due to persistent orthostatic intolerance (despite midodrine and fludrocortisone treatment and the use of “body support tights” up to the mamilla level). He worked half-time and had a child aged 10 years. No further medical complications had occurred.

The youngest brother (subject IV:3) followed a milder course and reported relative well-being during a telephone follow-up at age 35 years. He could tolerate upright posture without pharmacological treatment or compressive garments, but had a remaining tendency to presyncope and sometimes fainted. He worked full-time and was father of 2-year-old twins.

### Additional family members

Mean age at examination for all additional subjects of generation III and IV (8 subjects in total) was 64 years and 37 years, respectively. All subjects were tall, with the women having a mean length of 179 (range: 174–186) cm and the men having a mean length of 192 (range: 186–200) cm (compare with the average mean length in Swedes of 166 and 180 cm, respectively), and the mean BMI was 25.2 and 25.3 kg/m^2^, respectively. Five exhibited pes cavus (Table 4). Four subjects (III:2, IV:5, IV:6, III:4) had a history of previous or persisting tendency to syncope or presyncope. At examination, five subjects reacted with orthostatic BP fall and/or marked tachycardia when rising to upright position (Table 4). No one showed any clinical sign of polyneuropathy (criteria for normality: normal motor functions; no atrophy of the extensor digitorum brevis muscle; normal tendon reflexes; intact sensation in hands and feet for touch, vibration, and discrimination between sharp needle and blunt touch; detectable sweat moisture in palms and soles). The results suggested that one further, asymptomatic subject (III:3) shares the genotype (Table 4). A more detailed individual description of these family members is given in the ESM, file 1. Blood specimens for potential genetic diagnostics were obtained from all subjects.

**Table 4:** Blood pressure and heart rate reactions at supine rest and following rapid rise to upright position in additional members of the family

Subject, sex	Supine (BP; HR)^a^	Standing, immediately (BP; HR)	Standing, steady state 1 min (BP; HR)	Symptoms	Interpretation	Body feature	Conclusion
III:2, male	140/80 mm Hg; 72 bpm	133/80 mm Hg; 90 bpm	128/82 mm Hg; 82 bpm	–	Slightly marked sympathicotonic heart reaction	Tall	Affected?
III:3, female	150/95 mm Hg; 60 bpm	137/92 mm Hg; 75 bpm	145/90 mm Hg; 63 bpm	–	Normal reaction	Tall Pes cavus	Affected?^b^
IV:4, female	110/70 mm Hg; 65 bpm	115/72 mm Hg; 72 bpm	110/70 mm Hg; 72 bpm	–	Normal reaction	Tall	Normal
IV:5, female	115/72 mm Hg; 54 bpm	92/65 mm Hg; 90 bpm	110/75 mm Hg; 73 bpm	Dizziness	Marked sympathicotonic postural BP fall; persistent tachycardia at steady state	Very tall Pes cavus	Affected
IV:6, male	128/75 mm Hg; 55 bpm	115/70 mm Hg; 88 bpm	123/78 mm Hg; 78 bpm	Dizziness	Slight sympathicotonic postural BP fall; persistent tachycardia at steady state	Very tall Pes cavus	Affected
III:4, female	145/92 mm Hg; 71 bpm	125/82 mm Hg; 85 bpm	130/85 mm Hg; 83 bpm	Slight dizziness	Moderate sympathicotonic postural BP fall; mild persistent tachycardia at steady state	Pes cavus	Affected^c^
IV:7, female	120/70 mm Hg; 70 bpm	80/65 mm Hg; 110 bpm	105/72 mm Hg; 93 bpm	Dizziness	Marked sympathicotonic postural BP fall; persistent tachycardia at steady state	TallPes cavus	Affected
IV:8, male	122/77 mm Hg; 57 bpm	110/75 mm Hg; 72 bpm	113/70 mm Hg; 62 bpm	–	Normal reaction		Normal

Each value is mean of two consecutive measurements

See Fig. 1 for pedigree of the extended family demonstrating autosomal dominant OH

^a^Supine rest for at least 3 min preceded each uprise

^b^Note that daughter and son (IV:5, IV:6) of subject III:3 have overt symptoms

^c^Daughter (IV:7) of this subject has overt symptoms

## Discussion

We present here the results of a study on a two-generation family with members who segregate for profound OH with tachycardia and orthostatic intolerance. Four family members were thoroughly investigated: a father and two of his sons presented with orthostatic symptoms of grades 2 and 3–4, respectively [5], whereas a third son showed normal orthostatic tolerance but a greater than normal BP fall when standing. Investigations of additional close relatives revealed a total of eight–but likely ten (Fig. 1, Table 4)–affected family members, suggesting an autosomal dominant inheritance of the disorder. The majority of affected subjects were tall and slender with pes cavus, the latter making a hereditary polyneuropathy plausible, but clinical examination of all subjects and repeated neurophysiological examination of the symptomatic cases IV:1 and IV:3 showed intact peripheral nerve function.

### Pathophysiological aspects

#### Observations in the key family, cases IV:1, IV:3 and III:1

Several basic clinical observations in the thoroughly investigated cases, namely IV:1, IV:3 and III:1, support the presence of an intact sympathetic nervous system. The BP reactions to the Valsalva manoeuvre, apnoea and sustained handgrip, the electrodermal and skin vasoconstrictor responses, the results of test cycling in the lying position and the catecholamine levels were found to be normal in these subjects. The directly recorded sympathetic nerve activity in the index patient was qualitatively normal in terms of both muscle nerve fascicles (MSNA; baroreflex-governed activity involved in BP regulation) and skin nerve fascicles (SSNA), and showed normal responses to well-established manoeuvres (Valsalva, apnoea, sustained handgrip, arousal). Furthermore, the parasympathetic nervous system appeared to be normal, as deduced from normal RR-interval variation with breathing and from the bradycardia reaction with phenylephrine-induced rise in BP. There were no signs of abnormalities in heart function or heart sympathetic innervation, as evidenced by the immediate strong tachycardia following the BP fall when standing. A baroreceptor defect was contradicted by the orthostatic tachycardia response per se, by the suppression of MSNA after BP rise due to apnoea and Valsalva manoeuvre, the midodrine-induced bradycardia (Table 1) and the phenylephrine-induced bradycardia (Fig. 3). No sign of carotid hypersensitivity syndrome was seen. The cortisol axis and dopamine-β-hydroxylase metabolism [12, 29] were intact, with normal levels of noradrenalin in the blood and urine. A plausible arterial α-adrenoreceptor defect was excluded by the hypertensive response to phenylephrine infusion. Blood volume parameters were normal but, as illustrated by the effect of Ringer infusion, episodic dehydration and other deconditioning events may worsen the orthostatic intolerance, as observed in POTS [5].

Taken together, the present observations strongly suggest that the subjects suffer from blood pooling in lower body capacitance vessels with consequent insufficient venous return to the heart in the upright position. This conclusion is supported by the reduced preload at echocardiography in a sitting position, the flattened BP response during test cycling in the sitting (but not in the lying) position, as well as by the strong effect of crossing the legs when standing [24]. A suspected defect of the venous valve system of the legs could not be confirmed and no abnormal vasodilation tendency was observed with a metacholine test. Thus, with the investigative arsenal accessible, the exact mechanism behind the blood pooling could not be clarified. A selective impairment of lower body sympathetic venomotor function, suggested in POTS [4, 22, 30], cannot be ruled out, but neither is it demonstrated in the present family.

Venous pooling in the lower part of the body, suggested to occur in the present family members, is described to be one pathophysiological feature of POTS [5, 22, 30]. The clinical presentation of the present familial disorder differs from that of POTS, however, mainly due to the marked OH but also to the absence of non-cardiovascular symptoms associated with POTS [27, 31]. There are different opinions on whether ANS dysfunction is present in POTS [31, 32]; low-grade sympathetic denervation has been proposed to underlie POTS. In one study, MSNA was recorded in nine patients with POTS and nine matched controls [33]. Burst frequency was similar in both groups. During a hypotensive challenge (nitroprusside injection), the increase in burst frequency, but in not burst strength, was greater in patients than in controls. This was interpreted by the authors as a possible consequence of partial sympathetic denervation, i.e. reduced number of active sympathetic nerve fibres. A partial sympathetic denervation in the legs was concluded by the authors of another study, based on local noradrenaline spillover during certain manoeuvres [34]. These observations seem to be in opposition to our findings on the present index patient, who showed markedly increased burst strength in the peroneal nerve by 39% and 59% during Valsalva manoeuvre and apnoea, respectively, which speaks against a reduction in functioning sympathetic fibres. Likewise, direct recording of SSNA was of normal qualitative appearance and vivid (Fig. 2E), although a tentative reduction in the number of working sympathetic nerve fibres cannot be assessed with this method since the strength of nerve activity is strongly dependent on electrode position [15, 16]. The normal serum level of noradrenalin and its increase during standing also speak in favour of an intact ANS. To summarize, the present disorder shares some but not the main pathophysiological features with POTS.

The fall in BP in the present patients was immediate upon reaching an upright position, similar to that in “initial orthostatic hypotension” [7, 8]. This short-lasting reaction is thought to be caused by a temporal mismatch between cardiac output and vascular resistance with active rising. However, in the present cases this phenomenon is ruled out by its occurrence also at passive tilting and its persistence during a prolonged upright position.

The resting serum noradrenalin level, which was slightly above the reference interval in the index patient, seems to be in agreement with his relatively high burst frequency at rest. This allows no conclusion to be drawn due to the normal wide range of MNSA [35]; differences in the level of activity can be made only between relatively large groups of subjects. An “abnormally” high level of overall sympathetic activity at rest should be mirrored by clear-cut high serum noradrenalin levels in all key family members, which was not the case in this family. In principle, a very high burst frequency at rest means that a considerable degree of the maximum capacity is used already at rest, which theoretically might contribute to an insufficient BP defence with standing and other challenges to BP regulation, despite qualitatively normal appearance of MSNA at rest [26, 36, 37]. The burst frequency of the index patient is far from equal to such a situation, however.

Thus, the exact mechanism behind this disabling tendency to lower body venous accumulation of blood with postural load despite a seemingly intact ANS could not be revealed with the methods available.

#### Observations in the extended family study, generations III and IV.

Subject IV:5 suffered from repeated faints and displayed a marked postural BP fall with persistent relative tachycardia in the upright position. Subjects IV:6 and IV:7 displayed mild/moderate BP fall with strong sympathicotonic tachycardia reaction (per se indicating intact sympathetic innervation of the heart). Subject II:2 and III:4 displayed relatively normal BP reaction but somewhat exaggerated increase in heart rate.

Thus, these five relatives exhibit, to different degrees, the same phenotype as the index patient (subject IV:1), with the similarities, including body constitution. Subject III:3 reacted normally at examination, but she shares the body constitution with symptomatic subjects; she should be a gene carrier, being the mother of subjects IV:5 and IV:6.

### Genetics

The family comprises symptomatic subjects of both sexes and in at least two generations (Fig. 1), indicating an autosomal dominant disorder with variable expressivity of isolated OH. The phenotypic variation is marked, with subject IV:2 presenting with measurable but not symptomatic OH and subject IV:5 being markedly affected. Furthermore, the asymptomatic mother and grandfather of IV:5 (i.e. subjects III:3 and II:1) suggest a reduced penetrance if assuming that the orthostatic trait is inherited from the affected subject I:1.

Other phenotypic features vary as well, with the most affected subjects being tall and slender, with excavated feet; however, these features do not entirely co-segregate with OH.

The severe inherited disorder familial dysautonomia (HSAN III, Riley-Day syndrome) is excluded by the present individuals’ phenotype, and this conclusion is supported by the normal cardiac rhythm of MSNA, which is lost in familial dysautonomia [38]. Other rare forms of inherited and isolated OH have been associated with dopamine β-hydroxylase (*DHB*) deficiency or cytochrome b561 (*CYB561*) mutations transmitted as autosomal recessive traits [10, 12, 14]. Our exome sequencing of the most severely affected family member (subject IV:1) did not reveal any pathogenic gene variants in *DHB*, *CYB561* or in several other known genes associated with OH and catecholamine metabolism [13]. Given the dominant inheritance pattern in this family, the absence of *DHB* or *CYB561* mutations were expected as these are associated with recessive inheritance. However, autosomal dominant transmission of orthostatic intolerance has been reported previously [39], although in this earlier study the affected individuals presented also with additional and many faceted symptoms. Another report [40] presented five patients from four different and multiplex families with OH, tachycardia and blue-purple ankle discoloration and leg ecchymoses, i.e. with a partly different clinical presentation when compared to that of our family. A molecular genetic study on members of the latter four families suggested heterogeneity for a gene locus on chromosome 18q [41]. A later genome-wide scan of hypertensive siblings [42] suggested that genes on chromosome 18q might be involved in the regulation of systolic BP following a postural stressor, but the subjects were not selected on the basis of OH.

### Limitations of the study

The main shortcomings of the present study are: (1) that a pathophysiology underlying the tendency to blood pooling in the lower part of the body despite intact autonomic functions could not be established, and (2) a candidate gene responsible for the dysfunction is still to be determined. Due to the strong phenotypic variation in combination with the restricted number of symptomatic subjects (members in generation III and IV), further genetic analysis, such as segregation analysis, is not considered to be meaningful at the present time; if symptoms occur in the next generation, this may change.

### Conclusions

In conclusion, our study of this family with a phenomenologically unique disorder, with some affected family members being severely disabled, may uncover an as yet undescribed entity of OH transmitted as an autosomal dominant trait.

## Supplementary Information

Below is the link to the electronic supplementary material.

Supplementary file1 (DOCX 31 KB)

Supplementary file2 (XLSX 40 KB)

Supplementary file3 (XLSX 13 KB)

## Data Availability

The datasets generated and analyzed during the current study are available from the corresponding author on reasonable request.
