# Supplementary material for: Early-onset hereditary isolated non-neurogenic orthostatic hypotension in a Swedish family
Source: Clin Auton Res. 2023 Jul 17;33(4):421–32. doi: 10.1007/s10286-023-00963-9 (PMC10439023; doi:10.1007/s10286-023-00963-9)
Supplement: Supplementary file 1 — Supplementary file1 (DOCX 31 KB) [file 10286_2023_963_MOESM1_ESM.docx]

Early onset hereditary isolated non-neurogenic orthostatic hypotension in a Swedish family.

Jan Fagius, Joakim Klar, Niklas Dahl

**Supplementary material**

1. **Details on in main text briefly mentioned circulatory and chemical analyses with normal outcome, index patient IV:1.**

*Carotid massage*, performed manually, did not evoke a fall in BP.

*Plasma troponin-I* <0,022, normal.

*Myocardial perfusion scintigraphy* showed no obvious abnormality; LVEF 51 %, end-diastolic left ventricular volume 159 ml.

Succeeding *isotope angiography* confirmed normal function with LVEF at rest 51 % and during work load (supine, 100 W) 82 %.

*Continuous ECG for 48 hours*: no arrythmia. Average heart rate and HRV not commented in record report, but RR-interval variation during deep breathing and Valsalva manoeuvre was normal; see main text.

*Blood volume measurement* (^51^Cr-labelled autologous erythrocytes) was normal:

- erythrocyte volume) 32,5 ml/kg;
- total haemoglobin mass (THb) 10,7 g/kg;
- total blood volume (TBV) 77,7 ml/kg;
- plasma volume (PV) 45,2 ml/kg.

*Urine osmolality* (369 mOsm/kg) and *plasma renin level* (18,5 mIE/L) were normal.

*Diurnal cortisol curve* normal – peak morning level 482 nmol/L, afternoon nadir 95 nmol/L.

*Serum aldosterone* 165 nmol/L; ref <440.

*Serum level of dopamine* quoted normal in patient record, value lost.

*Urine excretion of catecholamines* normal – noradrenaline 6,8 nmol/h (ref <16,7), adrenaline 2,4 nmol/h (ref <3,8). *Serum adrenaline* normal, <0,3 nmol/L (ref <0,7).

1. **Clinical examination of close relatives outside the key family: history, symptoms and signs.**

**Subject III:2, male, age 68,** experienced in his teens clearcut but moderate tendency to presyncope, infrequently leading to syncope. Marked improvement with age. Otherwise healthy, no medication. Tall, length 191 cm, BMI 25,5; no pes cavus. No signs of polyneuropathy (criteria: normal motor functions; no atrophy of the extensor digitorum brevis muscle; normal tendon reflexes; intact sensation in hands and feet for touch, vibration, and discrimination between sharp needle and blunt touch; detectable sweat moisture in palms and soles).

**Subject III:3**, female, age 65. No history of disturbing orthostatic reaction. Medication for /supine/ hypertension and for rheumatoid arthritis since some years, otherwise healthy. Length 180 cm, BMI 27,8; somewhat high foot arches. No signs of polyneuropathy (see III:2 for definition).

**Subject IV:4**, female, age 38. No history of disturbing orthostatic reaction. Healthy, no medication. Length 176 cm, BMI 20,0; no pes cavus. No signs of polyneuropathy.

**Subject IV:5**, female, age 37. Strong tendency to presyncope and frequent syncope from the age of 10. Improvement with age but still marked lightheadedness in upright position and repeated fainting. Otherwise healthy, no medication. Very tall, 186 cm, BMI 21,7; high foot arches. No signs of polyneuropathy.

**Subject IV:6**, male, age 36. Clearcut but moderate tendency to presyncope from the age of 10; no faints. Still mild lightheadedness when rising up rapidly. Otherwise healthy, no medication. Very tall, 200 cm, BMI 23,5; high foot arches. No signs of polyneuropathy.

**Subject III:4**, female, age 60**.** Presyncope, in her teens,”must be cautious”. Still dizziness with rapid uprise from supine position. Medication for supine hypertension since a few years, otherwise healthy. Length 174 cm, BMI 25,8;, high foot arches. No signs of polyneuropathy.

**Subject IV:7**, female, age 39**.** No history of disturbing orthostatic reaction. Massive obesitas in her teens (”inheritance from father”); nowadays moderately so. Type 1-diabetes since age 19, insulin pump, well regulated; no known diabetic complications, no symptoms of polyneuropathy. Tall, 180 cm, moderately obese, BMI 30,9; high foot arches. No signs of polyneuropathy, clearcut hand and feet sweat moisture.

**Subject IV:8**, male, age 36. No history of disturbing orthostatic reactions. Healthy, no medicaton. Tall, 186 cm, BMI 26,9; no pes cavus. No signs of polyneuropathy.

Supplementary Tables S1 and S2 sent separately.
